# Site-Specific Neuromodulation of Detrusor and External Urethral Sphincter by Epidural Spinal Cord Stimulation

**DOI:** 10.3389/fnsys.2020.00047

**Published:** 2020-07-22

**Authors:** Yuriy Sysoev, Elena Bazhenova, Vsevolod Lyakhovetskii, Gleb Kovalev, Polina Shkorbatova, Regina Islamova, Natalia Pavlova, Oleg Gorskii, Natalia Merkulyeva, Dmitry Shkarupa, Pavel Musienko

**Affiliations:** ^1^Institute of Translational Biomedicine, Saint-Petersburg State University, Saint-Petersburg, Russia; ^2^Department of Pharmacology and Clinical Pharmacology, Saint-Petersburg State Chemical Pharmaceutical University, Saint-Petersburg, Russia; ^3^Pavlov Institute of Physiology, Russian Academy of Sciences (RAS), Saint-Petersburg, Russia; ^4^Granov Russian Research Center of Radiology and Surgical Technologies, Ministry of Healthcare of the Russian Federation, Saint-Petersburg, Russia; ^5^Clinic of High Medical Technology named after N.I. Pirogov St. Petersburg State University, Saint-Petersburg, Russia; ^6^Institute of Chemistry, Saint-Petersburg State University, Saint-Petersburg, Russia; ^7^Saint-Petersburg State Research Institute of Phthisiopulmonology, Ministry of Healthcare of the Russian Federation, Saint-Petersburg, Russia

**Keywords:** epidural spinal cord electrical stimulation, low urinary tract, external urethral sphincter, detrusor, neuromodulation

## Abstract

Impairments of the lower urinary tract function including urine storage and voiding are widely spread among patients with spinal cord injuries. The management of such patients includes bladder catheterization, surgical and pharmacological approaches, which reduce the morbidity from urinary tract-related complications. However, to date, there is no effective treatment of neurogenic bladder and restoration of urinary function. In the present study, we examined neuromodulation of detrusor (Detr) and external urethral sphincter by epidural electrical stimulation (EES) of lumbar and sacral regions of the spinal cord in chronic rats. To our knowledge, it is the first chronic study where detrusor and external urethral sphincter signals were recorded simultaneously to monitor their neuromodulation by site-specific spinal cord stimulation (SCS). The data obtained demonstrate that activation of detrusor muscle mainly occurs during the stimulation of the upper lumbar (L1) and lower lumbar (L5-L6) spinal segments whereas external urethral sphincter was activated predominantly by sacral stimulation. These findings can be used for the development of neurorehabilitation strategies based on spinal cord epidural stimulation for autonomic function recovery after severe spinal cord injury (SCI).

## Introduction

The abilities to store urine and control micturition are the principal functions of the lower urinary tract (LUT). LUT comprises two functionally different components: the bladder (detrusor) and urethra including internal and external urethral sphincters (EUS). In healthy rats, micturition involves simultaneous contraction of the detrusor (Detr), relaxation of the internal urethral sphincter (IUS), and bursting activity of EUS (Abud et al., 2015). These muscles work under the strict control of the cerebral cortex [right dorsolateral prefrontal cortex and the anterior cingulate gyrus (Blok et al., 1997, 1998)], pontine micturition center [also known as Barrington’s nucleus (Barrington, 1925)] and autonomic nervous system. Spinal cord injury (SCI) is often accompanied by disturbances of this hierarchy resulting in an overactive bladder, detrusor sphincter dyssynergia (DSD), or both (de Groat and Yoshimura, 2010).

Current treatments of neurogenic bladder and DSD may be divided into surgical and pharmacological approaches. The first one includes selective sacral rhizotomy which increases bladder capacity while preserving detrusor reflex and sphincter function (Rockswold et al., 1973) or a combination of sphincterotomy (Reynard et al., 2003) to decrease sphincter tone and enterocystoplasty, in which bladder capacity is increased by anastomosing a part of the ilium or ileocecal segment to the detrusor (Gurocak et al., 2007). However, sphincterotomy is largely supplanted by the use of botulinum toxin injections, medications, or urethral stents (Dorsher and McIntosh, 2012). Pharmacological treatments include anticholinergic (Wallis et al., 2016) or adrenergic medication (Welk et al., 2018) as a part of a comprehensive bladder management program. Despite the high prevalence of use, the beneficial effects of the above-mentioned options are limited due to low efficacy and side effects.

To date, several stimulation techniques, which can be used in combination with surgical and pharmacological approaches or alone, have been proposed. These include the direct stimulation of the bladder wall (Hald et al., 1967; Stenberg et al., 1967), stimulation of sacral (Li et al., 2016) or pudendal nerves (Vodušek et al., 1987; Previnaire et al., 1996; Hokanson et al., 2018; Li et al., 2018) and percutaneous (tibial) nerve stimulation (MacDiarmid et al., 2010; Peters et al., 2010, 2013). These approaches are quite effective in patients with LUT dysfunction, but there are several notable limitations. For example, direct bladder wall stimulation has had limited success in clinical practice due to a large number of electrodes and a high intensity of stimulation that is necessary. Clinical use has resulted in only local contractions of the bladder wall as well as unintended activation of the sphincter. Sacral nerve stimulation performed after the intradural approach is often associated with a high risk of mechanical damage (Rijkhoff et al., 1997). Tibial nerve stimulation requires intact supraspinal pathways and may not be suitable in patients with complete SCI, as was evidenced by animal studies (Xiao et al., 2014).

Future improvements for the treatment of LUT system disabilities might include neuromodulation of the spinal neuronal networks that contribute to the micturition control *via* epidural electrical stimulation (EES) of the spinal cord. Both animal and human studies have demonstrated that EES improves not only locomotor and postural functions (Minassian et al., 2004; Gerasimenko et al., 2008; Lavrov et al., 2015; Angeli et al., 2018; Gill et al., 2018) but also promotes the bladder control (Horst et al., 2011; Gad et al., 2014; Abud et al., 2015; Chang et al., 2018). However, the neuronal mechanisms underlying these effects have been poorly investigated. The main purpose of the present study was to reveal the effects of EES effects on the sympathetic, parasympathetic, and somatic networks that control the reflex activity of Detr and EUS. The obtained results expand our understanding of LUT spinal control and may result in the future development of rehabilitation algorithms for patients with SCI.

## Materials and Methods

The study was performed on four adult male Wistar rats (300–350 g body weight). All experimental procedures were approved by the Ethics Commission of the Pavlov Institute of Physiology. Experiments were performed in strong accordance with the requirements of Council Directive 2010/63EU of the European Parliament on the protection of animals used for experimental and other scientific purposes. The rats were housed in individual cages with free access to food and water. All surgical procedures were conducted under aseptic conditions under Isoflurane anesthesia (1%–2%;, mixed with Oxygen, a flow rate of 0.8 l/min).

The experiments were carried out with chronic implantation and testing during different time-points using the same EMG electrodes and electrodes for spinal cord stimulation (SCS). For chronic epidural electrodes implantation, partial laminectomies were performed and three Teflon-coated stainless steel wires (AS632, Cooner Wire, Chatsworth, CA, USA) from the Amphenol head connector cemented to the skull were passed under the vertebral arches inside the vertebral canal and above the dura mater of the remaining vertebrae between the partial laminectomy sites. Then the notch of insulation of 0.5 mm length was removed on each wire and the wires were sutured to the dura mater rostral and caudal to the exposed sites using 8.0 Ethilon suture. Then a midline lower abdominal incision was made to expose the bladder to implant the bladder catheter and stainless steel wire electrodes (AM-Systems, LLC, #793500) into the Detr, EUS (Scheepe et al., 1998; Merkulyeva et al., 2019). For surgical manipulations, the bladder was pulled out of the abdominal cavity, the access to the EUS was provided using a surgical dilator. The rostral portion of the pubic bone was partially removed using rongeurs to clearly expose the EUS muscle. The partially filled bladder was punctured by a needle (21G 0.8 × 40 mm) laterally on the left side and then a pre-marked catheter was inserted into the obtained hole so that its end was freely located in the cavity and did not touch the bladder walls. A plastic tube (Intramedic Polyethylene Tubing ID. 0.28 mm OD. 0.61 mm) conducted under the skin from the head to the bladder was used as a catheter that was implanted into the cavity of the bladder. The catheter was fixed in the bladder by using Ethilon 6.0 sutures. The further flow of fluid into the bladder through the catheter was provided using a cannula mounted on the free end of the catheter.

In addition to Detr and EUS, EMG electrodes were also implanted in gastrocnemius medialis (GM) and tibialis anterior (TA) muscles (Gerasimenko et al., 2006). In all cases, the needle and a small notch (~0.5 mm) were removed from the insulation of each wire to expose the conductor and form the electrodes. EMG electrodes were fixed together with Ethylon 4 suture at the entrance and exit from the muscle. Two common ground (indifferent EMG and stimulation grounds) wires (1 cm of the Teflon removed distally) were inserted subcutaneously in the mid-back region. All wires (for both EMG and epidural stimulation) were coiled in the back region to form a stress-release loop and were combined into one Amphenol head connector. The proper placement of the electrodes was verified during the surgery by stimulating through the head connector and post-mortem *via* dissection. Analgesia (ketorolac, 1 mg/kg, s/c) and antibiotic (enrofloxacin, 5 mg/kg, s/c) treatment were provided respectively 3 and 5 days after surgery. Bladder catheters were washed with distilled water once every two days during the experimental period.

After the testing of the reflex responses to SCS in 1 and 4 weeks after the initial bladder surgery, the lateral hemisection (van den Brand et al., 2012) at T8 spinal level was performed in each rat under gas anesthesia (isoflurane, 2–5%;). The spinal cord transection was verified by visual inspection under the microscope and then on the histological slices. Cut ends were exposed and separated by Gelfoam. An analgesic (ketorolac, 1 mg/kg, s/c) was given every 12 h for 48 h to relieve any post-operative pain. An antibiotic (enrofloxacin, 5 mg/kg, s/c) was given daily for 7 days to prevent urinary infection. Post-operatively, the bladder was manually expressed twice a day until the endpoint (1 week).

**Figure 1:**
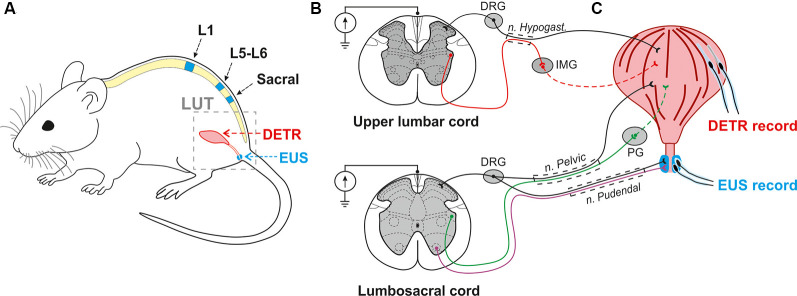
Experimental model to investigate the effect of epidural electrical stimulation (EES) to the lower urinary tract (LUT) system. **(A)** EES electrodes were placed over the upper lumbar (L1), lower lumbar (L5-L6), and sacral regions of the spinal cord. EMG electrodes were implanted in the external urethral sphincters (EUS) and Detrusor (Detr) muscles. **(B,C)** Associated LUT neuronal pathways activated by EES at the upper lumbar and lumbosacral cord. Coordinated activity of EUS and Detr muscles is provided by sympathetic, parasympathetic, and somatic projections from upper lumbar and lumbosacral regions of the spinal cord. Sympathetic pathways (red) from the upper lumbar cord to detrusor muscle course through the hypogastric nerve, inferior mesenteric ganglia (IMG), and postganglionic projections. Parasympathetic innervation (green) from the lumbosacral level of the spinal cord occurs *via* the pelvic nerve which extends fibers onto the postganglionic nerves through the pelvic ganglion (PG). EUS contractions are under the control of motoneurons (violet) originating from the Onuf’s nuclei situated in the ventral horns of spinal cord gray matter. Primary sensory neurons of dorsal root ganglia (DRG) carry sensory information from EUS and detrusor *via* hypogastric, pelvic, and pudendal nerves.

The main testing of the animals was performed in a chronic period (4 weeks) after bladder surgery. For supplementary experiments on the same group of animals, the additional analysis was done in an acute period (1 week) after the bladder surgery and soon (in 1 week) after the severe SCI (lateral hemisection). None of the implanted catheters or electrodes needed reimplantation. The reflex and urodynamic testing procedures were done on awake rats seated in the transparent plastic box with a cable from recording and stimulating equipment attached to the head plug. For urodynamic studies, the bladder catheters were connected to the infusion pump (ZooMed, SN-50C6). The infusion rate of the saline was 18 ml/h. In each rat, we analyzed the storage volume (volume of infused saline to start micturition) and the duration of the EUS bursting activity (Abud et al., 2015) during the micturition. For this, we performed 3–4 cycles of infusion/micturition. After the urodynamic recording, the motor evoked potentials were generated by EES (1 Hz frequency at stimulation intensities ranging from 50 μA to 800 μA in increments of 50 μA, 10 pulses for each stimulation amplitude, pulse duration of 0.2 ms) aiming to recruit various spinal pathways responsible for LUT and hindlimbs control (Figures 1A–C) in upper lumbar, lower lumbar and sacral spinal cord regions (Hou and Rabchevsky, 2014). The important criteria for the higher level of stimulation were to be in a painless range for the animals that was indicated by the calmness and immobility of the rats.

To trigger sympathetic pathways the upper stimulating electrode was implanted on the VT12 vertebral level and corresponded to L1, or border of L1–T13 spinal segments. The middle electrode was implanted on the VL1–2 vertebral level over the L5–L6 spinal region (Ishigooka et al., 2000) to stimulate a parasympathetic and somatic visceral network. The most caudal electrode was positioned on the VL2–VL3 vertebral level in relation to S3–S4 spinal segments, and spinal roots projecting afferent and efferent pathways from many overlying segments.

**Figure 2:**
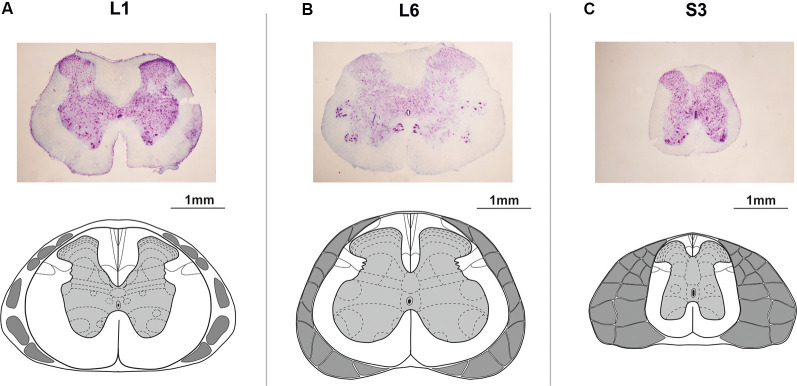
Histological microphotographs and corresponding schemes under the **(A)** upper lumbar (L1), **(B)** lower lumbar (L6), and **(C)** sacral (S3) electrodes. The schemes of the spinal segments and adjacent roots adapted from Watson et al. (2009).

At the end of experiments, animals were deeply anesthetized with an overdose of tiletamine-zolazepam (Virbac, France, 100 mg/kg, i/m) and then perfused transcardially with 0.9%; NaCl (150 ml), followed by 4%; paraformaldehyde (300 ml) in 0.1 M PBS, pH 7.4. Then a detailed dissection of vertebrae, roots, and spinal cord was performed to determine the exact level of the spinal cord stimulation. The lumbosacral cord was divided into segments based upon the grouping of the dorsal rootlets (Shkorbatova et al., 2019). To define the exact position of the epidural electrode, the dura mater below the electrode was marked with a permanent marker. After removing the dura mater, this mark was carefully transferred to the pia mater. Then the lumbosacral spinal cord was removed from the spine and stored in 20 and 30%; sucrose until it sank. The segments under the stimulating electrodes were cut on a freezing microtome into 50 mm transverse sections, stained with 4.1%; cresyl violet (Sigma–Aldrich, St. Louis, MO, USA) and compared with the spinal cord atlas (Watson et al., 2009) to verify the spinal cord level (Figures 2A–C).

**Figure 3:**
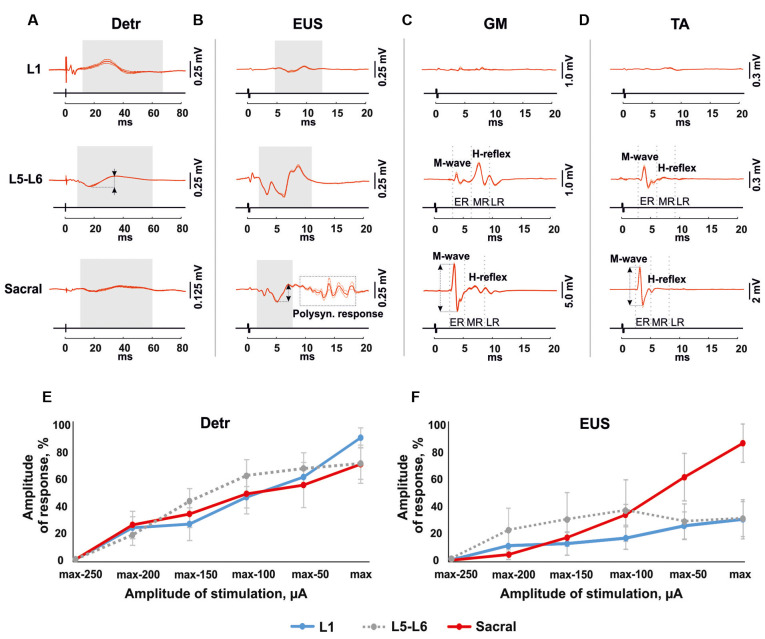
Mean evoked potentials from Detr **(A)**, EUS **(B)**, gastrocnemius medialis (GM) **(C)** and tibialis anterior (TA; **D**; *n* = 10 stimulation pulses at 1 Hz) during the EES at the maximal intensity of upper lumbar (L1), lower lumbar (L5-L6) and sacral spinal cord in 4 weeks after the surgery. The gray areas show the evoked potentials in Detr, EUS during spinal cord stimulation. The reflex activity in GM and TA was divided into the early response (ER), medium response (MR), and late response (LR) by gray dotted lines. The calculated latency of the responses corresponds to the left edge of the gray area for Detr and EUS, and the gray dotted line for an ER and MR of GM and TA. The amplitude of the response indicated as a two-way arrow between dashed lines. **(E,F)** Recruitment curves of normalized (*n* = 4 rats, mean ± SE) Detr and EUS responses during the upper lumbar, lower lumbar, and sacral stimulation.

The EMG signals were differentially amplified (A-M Systems USA, model 1700, the bandwidth of 10 Hz–5 kHz) and digitized at 20 kHz with a National Instrument A/D board. The reflex responses to spinal cord stimulation were recorded from Detr (Craggs and Stephenson, 1976; Fry et al., 1998) and EUS (Merkulyeva et al., 2019) while the tested awake rat was sitting in the plastic box. Also, we recorded GM and TA EMG activity to control the triggering capacity of EES and specificity of this method in recruiting spinal reflex pathways (Gerasimenko et al., 2006). For each stimulation amplitude, 10 responses were chosen for further analysis. Custom scripts written in Matlab were used to measure evoked potentials from the selected muscles. We analyzed latency and peak-to-peak amplitude of responses at the maximum intensity of stimulation (Figure 3). Since the LUT system function normally depends on the reciprocal activity of the Detr and EUS muscles, we measured the ratio of the Detr/EUS activation level (Figures 5B, 7C–E). The maximal amplitude of stimulation shown in (Figures 3E,F) was the same for both muscles in all stimulation points of one animal. Before averaging, each individual recruitment curve was normalized to the maximal response received in this animal either in the rostral or in medium or caudal stimulation points. All data are reported as mean ± SE. The hierarchical linear model with a constant slope and random intercept (Aarts et al., 2014) was used to compare latencies of Detr, EUS, GM and TA responses evoked by EES in rostral, medium and caudal points of the spinal cord stimulation, the volume and duration of voiding and the Detr/EUS and TA/GM activation level. The individual distributions of investigated values were normal in almost all cases by the Lilliefors test. The criterion level for the determination of statistical difference was set at *p* < 0.05.

**Figure 4:**
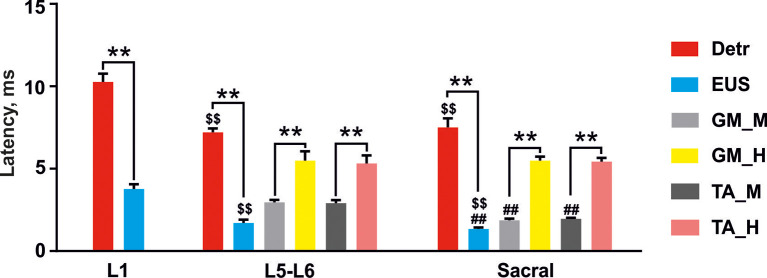
Latencies of the reflex responses in Detr, EUS, GM, and TA to stimulation of upper lumbar (L1), lower lumbar (L5–L6), and sacral spinal cord. The data is presented as mean ± SE (*n* = 4 rats, 10 stimuli per rat, mean ± SE). For the lower lumbar and sacral region the responses in GM and TA were divided into M-wave (GM_M and TA_M, gray and dark gray, respectively) and H-reflex (GM_H and TA_H, yellow and pink, respectively). Indication of significance level: **p < 0.01, ^\textdollar\textdollar^*p* < 0.01—vs. corresponding muscle response in upper lumbar region stimulation, ^##^*p* < 0.01—vs. corresponding muscle response in lower lumbar region stimulation.

**Figure 5:**
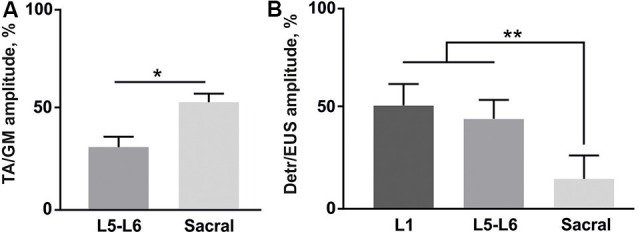
**(A)** Amplitude ratio of TA and GM activity during the stimulation of lower lumbar (L5-L6) and sacral spinal regions (*n* = 4 rats, 10 stimuli per rat, mean ± SE). **(B)** Amplitude ratio of Detr and EUS activity during the stimulation of three different regions of the spinal cord (*n* = 4 rats, 10 stimuli per rat, mean ± SE). Indication of significance level: *p < 0.05, **p < 0.01.

**Figure 6:**
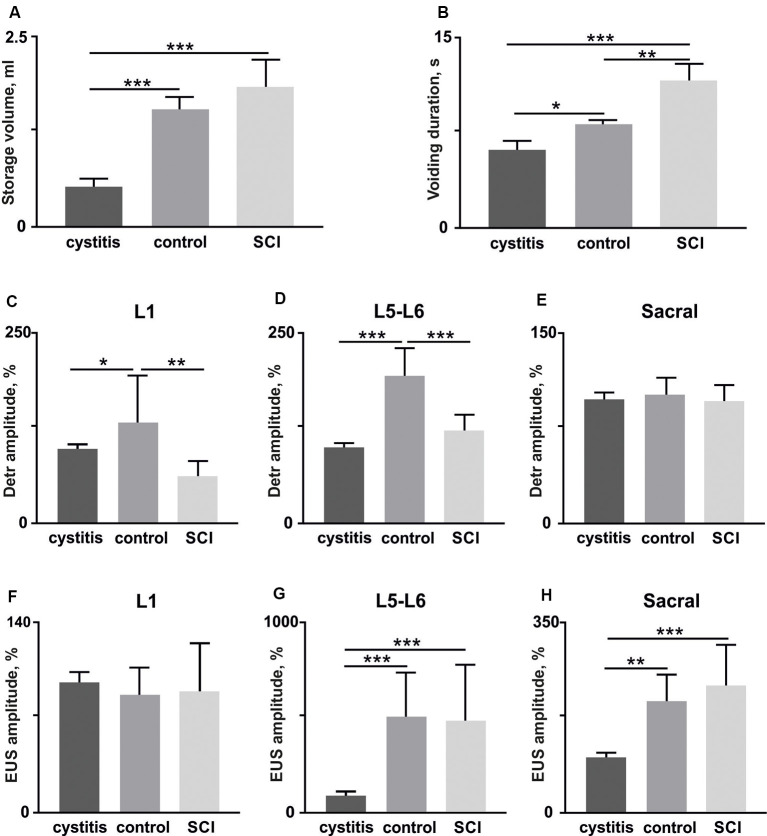
The volume of storage before the voiding **(A)** and voiding duration **(B)** in rats (*n* = 4) soon after (1 week) the bladder surgery (cystitis), after 4 weeks (control) and soon after (1 week) hemisection (spinal cord injury, SCI). **(C–E)** amplitudes of Detr muscle in upper lumbar, lower lumbar, and sacral segments (10 stimuli per rat, mean ± SE). **(F–H)** amplitudes of EUS muscle in upper lumbar, lower lumbar, and sacral segments (10 stimuli per rat, mean ± SE). The normalization was done per rat basis using amplitude in the acute period as 100%. Indication of significance level: *p < 0.05, **p < 0.01, ***p < 0.001.

**Figure 7:**
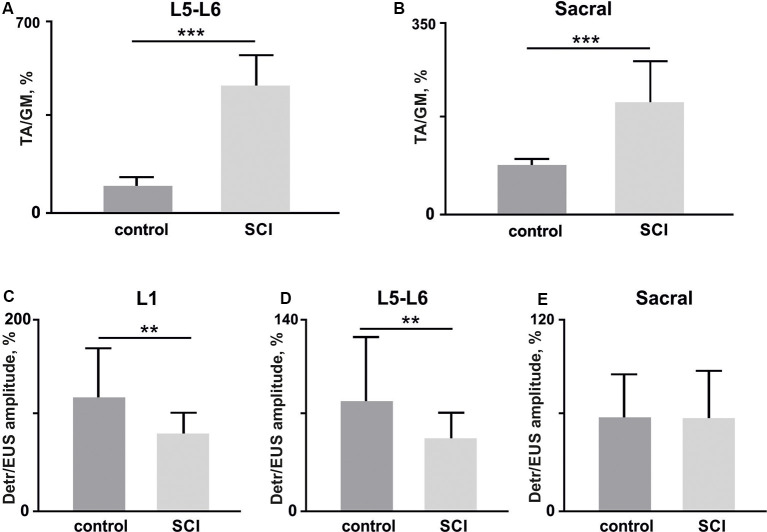
**(A,B)** The amplitude ratio of TA and GM muscles in lower lumbar (L5–L6, **(A)** and sacral **(B)** spinal cord stimulation (10 stimuli per rat, mean ± SE) 4 weeks after bladder surgery (control) and soon after (1 week) hemisection (SCI). **(C–E)** Amplitude ratio of Detr and EUS muscles in upper lumbar (L1, **C**), lower lumbar (L5–L6, **D**), and sacral **(E)** regions of the spinal cord (10 stimuli per rat, mean ± SE) in control and after SCI. Indication of significance level: **p < 0.01, ***p < 0.001.

## Results

We investigated the recruiting of different reflex pathways underlying Detr and EUS activity during EES of upper lumbar (L1), lower lumbar (L5–L6), and sacral spinal regions. We aimed to affect the sympathetic, parasympathetic, and somatic neuronal circuitry participating in the control of the LUT system.

### Effects of Upper Lumbar Spinal Stimulation on Detrusor and EUS Activity

Stimulation of the spinal cord in the upper lumbar region evoked responses in Detr and EUS muscles (Figure 3), whereas GM and TA responses were not present in all rats and were less prominent when extant. The latency of Detr responses was significantly longer (*p* < 0.01) than that of EUS (10.27 ± 0.50 and 3.77 ± 0.29 ms, respectively; Figures 3A–D, 4). The evoked potential of Detr muscle was represented by a slow wave of 20–40 ms duration that consisted of positive and negative peaks. EUS responses were relatively faster and shorter and could contain several positive and negative waves. In both muscles, the observed responses were stable and their amplitude gradually increased with rising the magnitude of stimulation (Figures 3E,F).

### Effects of Lower Lumbar Stimulation on Detrusor and EUS Activity

Unlike the upper lumbar region, the application of EES at the lower lumbar level-triggered responses in all recorded muscles. The Detr-evoked responses had the longest latency (7.21 ± 0.23 ms) and presented as a slow-wave composed of negative and positive components (Figures 3A, 4). Significantly, in this region of the GM and TA muscles, there was a well-defined division of responses (Figures 3C,D): (1) Early response (ER), or a direct motor axone M-wave (2.96 ± 0.14 and 2.91 ± 0.18 ms, for GM and TA, respectively); and (2) medium response (MR), or a primary afferents H-wave (5.49 ± 0.56 and 5.32 ± 0.48 ms, for GM and TA, respectively). These responses illustrated classical recruiting dynamics; the H-wave was suppressed by the M-wave (Hoffman, 1910), as the amplitude of EES increased (Gerasimenko et al., 2006). We could also observe the late reflex component (LR) in some of the animals but it was not consistent. The latencies of EUS (1.71 ± 0.20 ms), GM, and TA reflexes were significantly shorter (*p* < 0.01) than the Detr responses (Figures 3A–D, 4) and the general shape of EUS reflexes could contain several positive and negative peaks. Lower lumbar stimulation produced latencies in the Detr and EUS that were shorter (*p* < 0.01) than those observed during the upper lumbar stimulation (Figure 4). In the Detr and EUS muscles, the shape of the observed responses was rather stable, their amplitude increased as the stimulation magnitude rose until the submaximal level was saturated. Upon achievement of submaximal level, amplitude either reduced or remained unchanged up to the maximum level of EES (Figures 3E,F).

### Effects of Sacral Stimulation on Detrusor and EUS Activity

In contrast to the upper lumbar and lower lumbar regions, EES of the sacral level initially triggered responses only in the EUS, GM, and TA muscles. Only as stimulation intensity increased were responses also detected in the Detr muscle. The evoked potential in the Detr, similarly to other sites of EES, consisted of a slow wave with negative and positive peaks, whereas the EUS responses were fast and short, containing one or several positive and negative peaks. M-wave (1.87 ± 0.10 and 1.96 ± 0.06 ms, for GM and TA, respectively), and H-wave (5.49 ± 0.24 and 5.43 ± 0.23 ms, for GM and TA, respectively) for this region were similar to the lower lumbar (Figures 3C,D, 4). The high intensity (~from 450 μA and higher) stimulation-induced polysynaptic responses in EUS (~7–10 ms) in all (*n* = 4) animals (Figure 3B). The Detr responses had significantly (*p* < 0.01) longer latency (7.50 ± 0.56 ms) than either the short-term EUS responses (1.34 ± 0.09 ms) or the GM or TA M- and H-waves (Figure 4). All recorded muscles had significantly shorter (*p* < 0.01) latencies during stimulation of the sacral region than those of the upper and lower lumbar regions (Figure 4).

An overall review of EES recruiting Detr and EUS activity is presented in (Figures 3E,F). For both LUT muscles, an increase in upper lumbar and sacral stimulation led to a stable linear increase of the evoked responses, and no response saturation, even at the highest current values, was obtained (Figures 3E,F). For the EUS, though notably not for the Detr, sacral EES led to a response with a more developed amplitude increase of the response (Figure 3F).

Finally, we analyzed the amplitude ratio of Detr vs. EUS and flexor (TA) vs. extensor (GM; Figure 5). The flexor/extensor ratio analysis was done as a supplementary condition and compared the site-specific effects of the stimulation in low lumbar and sacral locations (*p* < 0.05) which confirmed the reliability of our approach (Figure 5A). Stimulation of the L5-L6 segment induced higher activity in GM, as expected due to the closeness of their motoneuronal pools and in contrast to the TA motoneurones, which are located 1–2 segments above (Capogrosso et al., 2013; Wenger et al., 2016. The Detr/EUS ratio was significantly (*p* < 0.01) lower in the sacral region than in the upper lumbar segment (29 ± 7%;) and lower lumbar region (43 ± 20%; Figure 5B). Altogether, these findings confirm that the pattern of Detr and EUS activity can be modulated through EUS activation by the EES of the sacral region and more pronounced Detr activity can be activated by stimulation of the L1 and L5–L6 regions.

### Relation of The Detr- and EUS-Evoked Potentials and The LUT Urodynamic Function

To show the functionality of the Detr- and EUS-evoked potentials (in the same group of rats, *n* = 4) we evaluated their relationship with the current functional state of the LUT system. To accomplish this, we performed two supplementary experiments with the impairments of the bladder, itself, and on supraspinal neuronal control.

First, we tested if detrusor and EUS reflex responses to EES related to the current condition of the LUT after bladder surgery. The reflex responses to stimulation of the different spinal cord regions 1 week after bladder surgery were compared with those present in the stabilized chronic period (4 weeks after surgery). A urodynamic study was performed to calculate the volume of storage before voiding and duration of voiding as general characteristics of the LUT system (Abud et al., 2015). We found, soon after the bladder surgery, urinary incontinence (Figures 6A,B), a lower volume of storage (Figure 6A), and corresponding reduced voiding duration (Figure 6B) as a manifestation of postoperative cystitis (Chang et al., 2019). Accordingly, we observed reduced Detr and EUS reflex responses 1 week after the bladder surgery. Note that significant and pronounced differences were found for the Detr during upper and lower lumbar SCS (Figures 6C–E), while for EUS—in lower and sacral SCS (Figures 6F–H). These results additionally support the importance of spinal region stimulation specificity concerning Detr and EUS activity.

Second, we impaired the supraspinal regulation of bladder control. A severe but incomplete SCI, lateral hemisection, induced motor deficiency in the hind limbs (Friedli et al., 2015), which was accompanied by relatively pronounced excitability of the flexor muscles and an increased ratio of TA/GM reflex responses amplitudes (Figures 7A–C). In addition to the reduction of predominant extensor activity after the SCI, we observed suppression of reflexes in the Detr when site-specific stimulation in upper and low lumbar spinal regions was applied (Figures 6C,D). This makes sense in the acute period after injury and is related to the bladder atony evidenced by an increase of the voiding duration (Figure 6B). These results are evidence of the fact that the Detr and EUS in upper and lower spinal SCS, are changed in the direction of detrusor’s excitability decrease (Figures 7C,D).

In sum, both supplementary experiments directly supported the reliability of the Detr and EUS reflex testing approach, reflecting that the current functional state of the LUT system depends on the Detr and EUS neuronal network excitability level.

## Discussion

### Neuromodulation of LUT System by Electrical Stimulation

Beneficial effects of EES to the LUT system, in combination with locomotor training after SCI, have previously been shown in both rats (Horst et al., 2011, 2013; Gad et al., 2014) and humans (Harkema et al., 2011). In the SCI rats study, it was observed that EES applied to the lumbosacral region of the spinal cord can facilitate the recovery of LUT function (Horst et al., 2011, 2013) and initiate micturition within seconds of stimulation onset (Gad et al., 2014). The authors proposed that EES enhances spinal neural networks excitability level (interneurons and motoneurons) and, when combined with motor training, increases the activation of the sensorimotor pathways that also control bladder function. In 2011, the first SCI patient exposed to EES demonstrated not only weight-bearing standing and some hindlimbs movements but also an ability to voluntarily void his bladder (Harkema et al., 2011). Further, recent studies have shown the efficiency of transcutaneous spinal cord stimulation in neuromodulation of LUT functions in rhesus monkeys (Gad et al., 2018a; Havton et al., 2019). Regarding SCI treatment in humans, it has been demonstrated that such non-invasive neuromodulatory techniques can normalize bladder and urethral sphincter function (Gad et al., 2018b; Herrity et al., 2018). However, while the proposed neuromodulatory treatments may be beneficial in some patients, in others it may be inefficient or cause unwanted side effects due to different integrity and excitability of spinal networks. Understanding the spatial distribution of neuronal projections that innervate the different LUT muscles can explain how best to apply SCS for maximal therapeutic efficacy.

In the present work, we show the site-specific effects of spinal cord stimulation to Detr and EUS activity and have proposed possible underlying reflex mechanisms of EES-mediated modulation of LUT functions. Testing the dynamics of Detr and EUS reflex activity in time after bladder surgery has shown that it was related to postoperative cystitis and recovery of the urodynamic function 4 weeks after. Moreover, we found the suppression of the evoked potentials in Detr muscle that matched bladder atony soon after the SCI. Therefore, similar to hindlimb motoneurons functional testing during EES (Lavrov et al., 2008), the evoked potentials in Detr and EUS muscles to spinal cord stimulation seems to reflect and can be used for the testing of LUT system functional state. Although future experiments that study the effects of different levels of EES to urodynamics are required, based on the data obtained, we suppose that the site-specific stimulation of the rostrocaudal visceral and spinal network can be an efficient form of therapeutic neuromodulation after the SCI and other diseases inducing LUT disorders.

### Neuronal Pathways Underlying The Reflex Activity of EUS and Detrusor Muscles Under EES

The data obtained show differences between detrusor and EUS activity in rats during the stimulation of three regions of the spinal cord. These results are in agreement with previous studies that proposed that the activity of detrusor and EUS occurs due to the activity of excitatory and inhibitory actions of a variety of segmental afferents, descending inputs, and sacral spinal actions (Shefchyk, 2001).

It is well known that detrusor muscle and EUS are controlled *via* parasympathetic, sympathetic, and somatic innervation in the lumbosacral regions of the spinal cord. In rats, parasympathetic nuclei situated in the lateral part of the spinal cord gray matter (L5-S1 level; Ishigooka et al., 2000; Hou and Rabchevsky, 2014) extend their axons to pelvic ganglia *via* the pelvic nerve (Figures 1B,C). The stimulatory action of acetylcholine (ACh), which is released from postganglionic nerve terminals on M3-muscarinic receptors induces bladder contraction (Lundberg, 1996) but causes simultaneous relaxation of urethral smooth muscles (IUS; Thornbury et al., 1992). Sympathetic nuclei of L1-L2 spinal cord level control LUT function through the hypogastric nerve and postganglionic projections. Norepinephrine released from postganglionic nerve terminals acts on β3-AR and causes bladder wall relaxation and, in direct contrast to ACh induces IUS contraction *via* α1-AR (de Groat et al., 1993; Andersson, 1999). Somatic innervation of EUS originating from the Onuf’s nuclei (L6-S1 level) controls the striated muscle contractions *via* the pudendal nerves (Drake et al., 2010).

Epidural stimulation of the spinal cord sympathetic region located in the L1 segment predominantly activated the detrusor muscle but not EUS (Figures 3E, 4B). This confirms the generally accepted view that hypogastric nerves and postganglionic projections innervate only the bladder wall and IUS, whereas EUS is controlled by the lower regions of the spinal cord (Hou and Rabchevsky, 2014). However, we also observed that stimulation of the upper lumbar region causes responses in the EUS with latency similar to GM (Figure 4A). It is plausible that the stimulation of the spinal cord upper lumbar region could trigger not only interneurons and motoneurons on this spinal level but also engage descending projections. These activities may be a part of propriospinal neuronal pathways or spinal-brainstem-spinal loop, for example, projections from L-region of Barrington’s nucleus which innervate sacral EUS motor neurons originating from Onuf’s nucleus (Morrison, 2008).

Independent of absolute amplitude values of EMG responses, the Detr and EUS ratios during L5–L6 stimulation were higher than during sacral stimulation (Figure 4B). This indicates that EES of the lower lumbar region had a more facilitating effect to detrusor muscle than did sacral EES. Since the stimulating electrode is positioned close to the detrusor parasympathetic preganglionic neurons and EUS motoneurons, the electrical current directly recruits two subsystems that have “competitive” reflex mechanisms (Shefchyk, 2001). Perhaps this causes the saturation effect, which we observed in Detr and EUS when stimulating lower lumbar segments at maximal magnitudes (Figures 3D,E).

Opposite results were obtained during the EES of the sacral region; we found higher activation of EUS than of the detrusor (Figures 3D, 4B). Due to the anatomy of the spinal neuronal pathways under the sacral electrode, we recruited the roots from the majority of lumbar and sacral segments (Figure 2C). Most fibers of these roots carry sensorimotor information and form the peripheral nerves of the hind limbs. EUS activation during sacral stimulation can be associated with the somatovisceral integrative mechanisms (Merkulyeva et al., 2019). This effect is similar to tibial nerve stimulation, which is known to be effective in the clinical practice for EUS activation and treatment of the overactive bladder syndrome (Peters et al., 2010, 2013).

To confirm that the testing protocol of the reflex responses recruiting is well established, as a control experiment, we recorded an EES-evoked reflex activity of well studied GM and TA muscles. Similar to previous work (Gerasimenko et al., 2006), we obtained pronounced dynamics of H-reflex and M-wave on the recruitment curve of GM and TA muscles when the lower lumbar and sacral regions were stimulated. The H-reflex is associated with stimulation of group Ia afferents that project monosynaptically to motoneurons, whereas M-wave is a direct motor response due to stimulation of motor axons (Knikou, 2008) that project into the spinal roots from overlying segments (Figure 2C). It is worthwhile to note that, in some rats, we observed similar H/M-dynamic as in the EUS, but this phenomenon was not pronounced. It can be assumed that the EUS short-latency evoked potentials that have a similar nature with M-wave of GM and TA i.e., direct excitation of appropriate motor neurons. However, we do not deny that earlier recruiting responses of EUS with a latency of ~4 ms can be an H-reflex, caused by activation of Ia afferents from muscle spindles. The existence of rare muscle spindles in human EUS was shown by Lassmann (1984). Significant differences between latencies of EUS and GM or TA responses (Figure 4A) may be related to different path lengths from the spinal cord stimulation area to EUS and GM or TA muscles, respectively.

### Site-Specific Activation of The Spinal Network to Recover Visceral Function After SCI

Even though SCI disturbs spinal reflexes the lumbosacral mechanisms which remain intact provide an opportunity for restoration of LUT functions. In this article, we have shown that directly-applied EES can modulate EUS and detrusor reflex activity. Although further investigation of the EES effects on the urodynamic activity of the Detr and EUS is required, this may be a promising tool for the treatment of LUT disturbances manifesting as an inability to store and expel urine. Besides the short-latency reflex response, we have also observed polysynaptic activity recruited by EES in Detr and EUS that is apparently due to activation of visceral and sensorimotor neuronal pathways underlying somatovisceral integrative mechanisms (Merkulyeva et al., 2019). Such mechanisms can be essential for the motor and autonomic functions recovery after SCI.

Site-specific modulation of EUS activity had previously been reported in rats with spinal cord and peripheral nerve injury (Abud et al., 2015; Chang et al., 2018). This is based on the evidence of the EUS-associated spinal neuronal network distribution in the thoracolumbar cord, the circuitry that controls tonic activity at L6–S1, and bursting activity between T8 and T9 and L3 and L4 (Chang et al., 2007). It was later confirmed that EES of predominantly caudal lumbar segments triggers EUS tonic contractions whereas stimulation of upper lumbar segments inhibits EUS tonic activity and elicited EUS bursting (Abud et al., 2015; Chang et al., 2018). Following our results, it was shown, in monkeys, that bladder pressure responses to spinal cord stimulation are mainly triggered by upper lumbar cord whereas EUS responses predominantly occurred due to lumbosacral enlargement of the lower regions (Gad et al., 2018a).

Recent clinical study of Kreydin et al. indicated that in patients with different pathologies (SCI, stroke, MS or idiopathic overactive bladder) stimulation of the spinal cord decreased detrusor overactivity, improved continence, and enhanced LUT sensation (Kreydin et al., 2020). Taken together with our data, these results provide a rationale for the development of spinal neuroprostheses that would enable to control LUT functions by the spatiotemporal neuromodulation approach (Wenger et al., 2016) that could be optimized to the specific clinical situation. In the recent study by Herrity et al. (2018), Medtronic implantable neurostimulation interface was used for bladder mapping. Effective configuration and stimulation parameters were successfully applied to improve reflex voiding efficiency in SCI patients. The bladder dysfunction caused by SCI often changes over the course of the injury. For instance, from bladder atonia to an overactive bladder (Cruz and Cruz, 2011). So, the possibility to change not only parameters of electrical current but also the site of stimulation is a very useful option. To date several neuroprosthetic arrays have been proposed (Borton et al., 2014; Hahnewald et al., 2016; Minev et al., 2015; Bareket et al., 2017); there are several existing design solutions (i.e., by Medtronic, Boston Scientific), including LUT spinal implants. Application of these spinal neuroprostheses is non restricted to SCI and may be suitable in patients with other neurological conditions that are accompanied by LUT dysfunction, for example, multiple sclerosis (Phé et al., 2016; Peyronnet et al., 2019) or Parkinson’s disease (Winge, 2015; Hajebrahimi et al., 2019).

## Conclusions

The data obtained demonstrate the neuromodulation of the LUT system by EES of lumbar and sacral regions of the spinal cord in chronic rats. The detrusor muscle activation mainly occurs during the stimulation of the upper L1 and lower lumbar (L5–L6) spinal segments whereas EUS was activated predominantly by sacral stimulation. These findings can be used for the development of neurorehabilitation strategies based on SCS for impaired autonomic function recovery.

## Data Availability Statement

The raw data supporting the conclusions of this article will be made available by the authors, without undue reservation.

## Ethics Statement

The animal study was reviewed and approved by Ethics Commission of the Pavlov Institute of Physiology.

## Author Contributions

PM conceived the experiments. EB, YS, NP, GK, NM, OG and PM designed and performed the research. YS, VL, PS and PM analyzed the data. YS and PM wrote the article. YS, NM, PS, VL, DS, RI and PM edited the article. PM supervised the study.

## Conflict of Interest

The authors declare that the research was conducted in the absence of any commercial or financial relationships that could be construed as a potential conflict of interest.
